# The core vocabulary of South African Afrikaans-speaking Grade R learners without disabilities

**DOI:** 10.4102/sajcd.v67i1.701

**Published:** 2020-07-20

**Authors:** Danél Hattingh, Kerstin M. Tönsing

**Affiliations:** 1 Centre for Augmentative and Alternative Communication, University of Pretoria, Pretoria, South Africa

**Keywords:** Afrikaans, augmentative and alternative communication (AAC), core vocabulary, Grade R learners, vocabulary selection

## Abstract

**Background:**

Augmentative and alternative communication (AAC) can enable individuals with little or no functional speech to communicate functionally in a variety of communication contexts. AAC systems for individuals who are not (yet) fully literate often require that the vocabulary for the system be preselected. By including the most commonly- and most frequently-used words (core vocabulary) in an AAC system, access to novel utterance generation can arguably be facilitated. At present, no Afrikaans core vocabulary list based on children’s speech samples exists.

**Objectives:**

This study aimed to identify the most frequently- and commonly-used words of South African Afrikaans-speaking Grade R learners without disabilities.

**Method:**

Spontaneous speech samples were collected from 12 Afrikaans-speaking Grade R learners during regular preschool activities. Samples were transcribed and analysed to determine the number of different words used, the frequency with which each word was used, as well as the commonality of word use across the 12 participants.

**Results:**

A total of 239 words met the criteria for inclusion in the core vocabulary (words used with a frequency of more than 0.05% in the sample, and used by at least half of the participants). These words accounted for 79.4% of words used in the entire speech sample.

**Conclusion:**

The established core vocabulary consists of a relatively small set of words that was found to represent a large proportion of speech. AAC team members may consider including these words on Afrikaans AAC systems that are intended to give access to a measure of novel utterance generation.

## Introduction

Augmentative and alternative communication (AAC) systems, such as those representing words and messages with graphic symbols, can support communication for persons with little or no functional speech who are not (yet) fully literate. Graphic symbol-based systems require the preselection of vocabulary. The selected vocabulary should enable the person using the system to meet all their communication needs across different contexts and with different partners (Fallon, Light & Paige, 2001), but should be small enough to minimise the cognitive and motor demands in memorising and navigating to the location of the vocabulary. Vocabulary selection typically requires people other than the individual who will use the vocabulary to predict the words this person will require in all the communication situations that he or she encounters (Dark & Balandin, 2007). This is a complex process, often leading to the inclusion of irrelevant and seldom-used words (Dark & Balandin, 2007). Various ways to select the most relevant and appropriate vocabulary have been suggested and researched (Thistle & Wilkinson, 2015). One such method is the use of core vocabulary lists as a resource for vocabulary selection.

Previous studies in English and other European languages have indicated that 200–250 spoken words account for approximately 80% of a person’s spoken communication (Robillard, Mayer-Crittenden, Minor-Corriveau & Bélanger, 2014; Trembath, Balandin, & Togher, 2007). Spoken language sample analyses in Korean, Mandarin Chinese and isiZulu have also indicated that it is possible to isolate a limited number of lexical items (although these may not always be words but may also include morphemes) that are used with a high frequency and are reused continuously in conversations (Liu & Sloane, 2006; Mngomezulu, Tönsing, Dada & Bokaba, 2019; Shin & Hill, 2016). These frequently- and commonly-used words/morphemes have been termed ‘core vocabulary’. Core vocabulary may be useful for inclusion on AAC systems because of its small size but high potential for reusability across contexts and partners (Baker & Chang, 2006). Core vocabularies typically contain a number of structure words – words that have little semantic meaning but perform a grammatical function and therefore contribute to the grammatical correctness of sentences (Witkowski & Baker, 2012). Certain parts of speech, such as conjunctions and prepositions, are typically classified as structure words. Content words, such as nouns, verbs and adjectives, on the other hand, do have a semantic meaning.

Although Afrikaans vocabulary frequency lists have been published, these are either derived from written sources or the source of origin is not mentioned (Barnes, 1995; Malan, 1943). Since written language samples have been shown to differ from spoken language samples, these lists may not be completely representative of the spoken core vocabulary used by children (Liu & Sloane, 2006). Similarly, the translation of existing core vocabulary lists derived from samples in languages other than Afrikaans is also unlikely to yield an appropriate representative core vocabulary set, because morphological differences between different languages result in (at times substantial) differences in vocabulary lists of specific languages (Mngomezulu et al., 2019). These morphological differences would affect especially structure vocabulary. Sociocultural and geographic influences have also been noted in the core vocabulary, affecting specifically nouns, slang words and interjections (Balandin & Iacono, 1999). These differences have been noted even between Australian and American English adult-based core vocabulary lists, and can therefore be expected to be found in the lists generated for different languages. Language-specific frequency lists that are based on original spoken language samples are therefore required.

The main aim of this study was to determine the Afrikaans core vocabulary of Grade R learners without disabilities. The study had the following objectives: (1) to analyse language samples of Afrikaans-speaking Grade R learners obtained during regular preschool activities in order to determine the total number of words, the number of different/unique words (NDW), and the most frequently- and commonly-used words (core vocabulary); and (2) to further describe the core vocabulary by classifying words into content and structure words.

## Method

### Design

A quantitative non-experimental descriptive observational design was used in this study (McMillan & Schumacher, 2010). Audio recordings were made of participants’ speech and these were analysed to determine the core vocabulary.

### Setting

The participants for the study were recruited from five Grade R classes from three preschools with Afrikaans as the primary language of instruction in a metropolitan area. All three schools were located in a middle- to high-income area. Classes ranged in size from 15 to 29 children, with an adult:child ratio ranging from 1:12 to 1:15.

### Participants

Convenience sampling was used to identify five Grade R classes where Afrikaans was the language of teaching and learning, located within a reasonable travel distance from the researcher’s residence. Class teachers were requested to nominate between two and four learners per class (equal numbers of boys and girls per class) for possible inclusion in the study. A total of 12 Grade R learners aged 5;2 (years;months) to 6;10 were included. The specific age range was chosen as most children with typical language development of this age use adult-like language structures (Owens, 2012). In addition, children had to meet the following selection criteria in order to be included: (1) Afrikaans home language; (2) no concerns about language development, and no history of language impairment or delay; (3) no concerns about general development; (4) attendance of the preschool for at least 2 months prior to data collection, and weekly attendance of at least twice a week. Questionnaires completed by parents and teachers were used to ensure that children met the selection criteria (Hattingh, 2018).

### Data collection

Participants wore digital audio-recording devices in small zipped pouches around their waist, with lapel microphones attached to the collars of their clothing. The devices, which were fitted and turned on at the beginning of the school day, recorded the participants’ speech during regular preschool activities, such as book reading and free-play. Teachers could switch off and/or remove and could refit/turn on the devices again at their own discretion. Recordings continued on a daily basis until 3500 words (including unintelligible words and utterances) had been recorded per participant. The time taken to reach this number of words varied from 2 h 16 min to 6 h 50 min, recorded across 1–3 days.

### Data analysis

The first 20 min of the recordings were discarded to limit novelty effects. Any subsequent utterances referencing to the equipment or data collection process were also excluded from the transcriptions, in order to ensure that the speech samples remained as authentic as possible (Trembath et al., 2007). The rest of the recordings were transcribed verbatim by the first author into the *Systematic Analysis of Language Transcripts* software (Miller & Iglesias, 2012), using relevant transcription rules (Hattingh, 2018). Additional codes were added to the words in the transcription to ensure that inflectional morphological variations of different parts of speech were traceable to the uninflected root form of the word.

A second transcriber transcribed a randomly-selected segment of recording per participant, amounting to 20% of the total recording (Ayres & Ledford, 2014). The transcription was compared to that of the researcher, and word-by-word agreement was calculated by dividing the number of identically transcribed words by the sum of words transcribed identically, words omitted, words added and words transcribed differently. The percentage of agreement obtained in the study was 80%. Although at the lower end of acceptability, this percentage seems common for studies where real-time conversations are recorded (Mngomezulu et al., 2019; Robillard et al., 2014). The second transcriber also independently coded 20% of the transcriptions, and this coding was compared to the coding done by the researcher. Percentage agreement was found to be 82%. The Systematic Analysis of Language Transcripts program was used to calculate the total number of words, NDW, as well as the type-token ratio (TTR). Unintelligible words were excluded from the analysis.

The transcriptions did not make distinctions between words with the same orthographic forms but two or more distinct meanings (homonyms) or functions (polysemes). Therefore, core vocabulary needed to be determined in three steps. In Step 1, the frequency and commonality criteria were applied, and all words that occurred with a frequency of at least 0.05 per 100 words (i.e., 0.05% or once in every 2000 words) and that were used by at least six or 50% of the participants (i.e., commonality score of 6) were listed. Step 2 involved looking up all words in this list in the dictionary (Odendal & Gouws, 2012), to determine if they had more than one meaning and/or grammatical function. Where this was the case, words in the transcripts were given unique codes to separate them by meaning/function, leading to the recalculation of frequency and commonality scores for the respective forms. During Step 3, frequency and commonality scores were re-examined to ensure that words that were separated into their respective meanings/functions complied with the set criteria described in Step 1.

### Ethical consideration

Prior to data collection, the study was approved by the Research Ethics Committee of the Faculty of Humanities of the University of Pretoria (protocol number GW20171002HS). Permission from the Gauteng Department of Education, and the principals of the relevant preschools were also obtained. Furthermore, parents/legal guardians were informed of all aspects of the study via an information letter and their written consent was obtained prior to approaching the children and providing them with verbal information on all aspects of the study using a script in child-friendly language and pictures to support comprehension. Only children who assented to participate were included in the study. Ongoing verbal assent was obtained every time the participant was fitted with the audio recorder. All ethical principles regarding the involvement of human participants as set out in the Belmont report (Department of Health Education and Welfare, 1979) were adhered to in the study.

## Results

After unintelligible units were excluded, the words collected per participant ranged from 3108 to 3419 words. The composite sample across all 12 participants consisted of 39 645 orthographic words. The NDW obtained from the composite sample amounted to 3304 orthographically distinct words, with an overall TTR of 0.08 or 1:12.

A core vocabulary list consisting of 239 words was determined (see Appendix 1) by using the three-step process described in the methods section. The average commonality score of the 239 core words was 10. The remaining words made up the fringe vocabulary. Core words had a coverage of 79.4% of the entire composite sample. This means that, of the 39 645 orthographic words that made up the composite sample, 79.4% were core words. Fringe words therefore covered the remaining 20.6% of the sample.

The 239 words identified as core vocabulary were categorised into two broad categories consisting of content words (carrying a semantic meaning) and structure words (words that have little semantic meaning and perform a grammatical function) (Van Rooy, 2017). Of the 239 words in the vocabulary list, 76 were structure words and 163 were content words.

**FIGURE 1:**
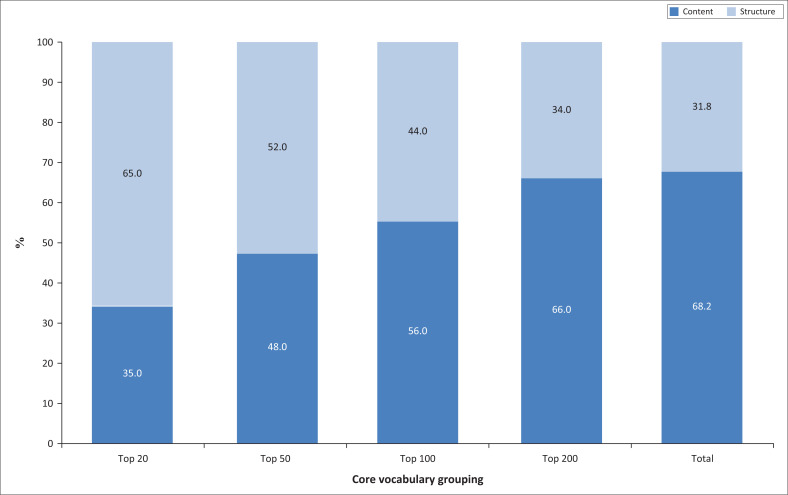
Percentage of content versus structure words in the top 20, 50, 100, 200 and total core vocabulary words.

The percentage of content versus structure words in the most frequently-used 20, 50, 100, 200 and total number of core words is provided in Figure 1. From the figure it can be seen that the percentage of content words increases as the number of words increases.

Although there were more content than structure words (in number) in the core vocabulary, content words were used only marginally more often than structure words. Whilst the total coverage of the core was 79.4%, 41.0% of words used during conversations were content core words, whilst 38.4% of words were structure core words. The TTR for content words was about 0.01 (or 1:100), whilst the TTR for structure words was about 0.005 (or 1:200). This means that, on average, a content word occurred 100 times in the sample, whilst a structure word occurred 200 times on average.

The core vocabulary was categorised into 14 different parts of speech. Results are represented in Table 1. Verbs had the highest NDW (*n* = 53), followed by adverbs (*n* = 31) and nouns and pronouns (*n* = 29 each). The categories miscellaneous words (*n* = 1), articles (*n* = 2) and enclitic words (*n* = 2) had the lowest NDW. Regarding frequency of use, the part of speech most frequently used was pronouns, constituting 20.2% of the total sample. This was followed by verbs and adverbs, representing 18.3% and 11.3% of the total sample, respectively. The NDW contained per category does not necessarily predict the frequency of use of that category. For example, 29 pronouns covered 20.2% of the sample, whereas 53 verbs covered 18.3%. The category ‘articles’, on the contrary, contained only two words (*’n* and *die*), but accounted for 4.0% of the sample.

**TABLE 1:** Core words classified according to parts of speech.

Parts of speech	NDW	Proportion (in terms of NDW) in core (%)	Number of occurrences in the sample	Frequency of occurrence (%)
Pronouns	29	12.1	8013	20.2
Verbs	53	22.2	7252	18.3
Adverbs	31	13.0	4482	11.3
Interjections	22	9.2	2345	5.9
Conjunctions	9	3.8	1644	4.2
Articles	2	0.8	1586	4.0
Nouns	29	12.1	1362	3.4
Proper nouns†	3	1.3	1178	3.0
Prepositions	10	4.2	1138	2.9
Adjectives	21	8.8	960	2.4
Code switches	18	7.5	675	1.7
Numerals	9	3.8	499	1.3
Enclitics	2	0.8	337	0.8
Miscellaneous	1	0.4	22	0.06
**Total**	**239**	**100**	**31 493**	**79.4**

NDW, the number of different/unique words; CN, Child Name; TN, Teacher Name; AN, Adult Name.

† , The codes that were used, CN, TN and AN, were not differentiated. This might have led to an over-representation of proper names.

## Discussion

The language sample of 39 645 words obtained in this study yielded 3304 unique words (NDW). The core vocabulary established by the three-step process consisted of 239 words, which accounted for 79.4% of the composite sample. The results of the study show vocabulary frequency patterns that are in many ways similar to those found in studies done in other languages, where the total number of words or linguistic units used typically outnumbered the unique number of words or linguistic units by a considerable margin (Boenisch, 2014; Liu & Sloane, 2006). As reported in other studies, a considerable proportion of the total words is represented by a relatively small set of unique words. These results are consistent with those from previous literature, indicating that a small set of linguistic units are repeatedly reused with a high frequency during conversations.

The proportion of structure versus content words in the Afrikaans core vocabulary (which amounted to 32% and 68% respectively) was found to be identical to that found in the English core vocabulary established by Boenisch and Soto (2015). These authors found that in the top 300 words used most frequently by native English speakers, 68% were designated as content vocabulary, whereas 32% were designated as structure vocabulary. A similar pattern was also discerned by Mngomezulu et al. (2019), who found that 68% of the isiZulu core vocabulary established, consisted of content formatives, whereas 29% consisted of structure formatives (with 3% consisting of words classified neither as content nor core).

Although there were less structure words than content words in the core vocabulary, the coverage of content and structure core words differed only marginally. The TTRs show that, on average, a structure core word occurred twice as often as a content core word in the sample. Mngomezulu et al. (2019) also found that there were more content than structure formatives in the isiZulu core vocabulary, but that structure formatives occurred with a higher frequency. In their study, structure formatives occurred about 3.3 times as often (on average) as content formatives.

Similarities were found in terms of the parts of speech making up the core vocabulary. Pronouns, verbs and adverbs made up a large proportion of the core vocabulary in this and other studies (Boenisch & Soto, 2015; Mngomezulu et al., 2019). These parts of speech tended to be used with a high frequency; for example, many different verbs were found in the core vocabulary whilst also being used with a high frequency. Similarly, pronouns and adverbs featured prominently, both in terms of NDW and in terms of frequency of use. These findings suggest some similarities in the way parts of speech are used across languages. Verbs, for example, seem to be central to sentence construction across languages (Sutton, Soto & Blockberger, 2002).

## Strengths and limitations

The Afrikaans core vocabulary established in this study is based on language samples collected across a variety of preschool activities, resulting in a vocabulary that is not limited to, for example, playtime or snack time. Furthermore, excluding the first 20 min of each sample reduced the novelty effect that the recorders might have had on the participants’ speech. This study is one of few where homonyms and polysemes were rigorously separated. The aim thereof was to ensure that the meaning of each word in the core vocabulary list was unambiguous, thus enabling appropriate graphic representation of the words in an AAC system.

Whilst the inflected morphological variations of nouns, verbs, numerals, pronouns and adjectives were counted together under the root form of the word in this study, the codes and tags added to the transcription also allowed for frequency information for each variation to be obtained (Hattingh, 2018). This information can assist practitioners to make informed decisions about the necessity of including access to inflected forms on specific AAC systems.

The relatively small sample size in combination with participants from similar ages across three fairly similar sites, as well as the limited time span taken for data collection, influenced the generalisability of the data. The criteria for determining core vocabulary consisting of frequency and commonality scores have also been criticised as arbitrary (Shin & Hill, 2016). In the present study, vocabulary was included based on a commonality score of at least six (i.e., at least 50%) and a frequency count of equal to or more than 0.05%. There is no scientific reason why words with a frequency of 0.05% are designated as core vocabulary, whilst words with a frequency count of 0.049% are designated as fringe. The commonality criterion is equally arbitrary. Shin and Hill (2016) suggest that grouped frequency counts are a more rigorous and defensible way to distinguish between core and fringe words.

## Implications for practice and further research

The established Afrikaans core vocabulary list (Hattingh, 2018) can be used as a vocabulary source for Afrikaans-speaking children who require AAC. Words can be judiciously selected from this list for inclusion on AAC systems. The core word list can be particularly helpful to alert team members to the importance of including structure words, since these words provide access to morphology and syntax. However, the provided core list is not intended to be used in isolation, but in combination with a child-specific fringe vocabulary. Environmental inventories and informants can assist in determining these fringe words that should reflect the interests, personality, and contexts of the child in need of the system (Robillard et al., 2014). An appropriate combination of core and fringe vocabulary should allow children to express themselves appropriately in various communication situations.

Since this is the first core word list established in Afrikaans based on a sample of spoken words, clinicians may also consider referring to it when selecting words for Afrikaans individuals of other ages or for use across other settings, as previous research has indicated that core vocabularies are, to some extent, useful across settings and individuals (Van Tilborg & Deckers, 2016). However, this should be done judiciously, as certain words in the core vocabulary list (specifically nouns and verbs such as *blok* [‘block’] and *inkleur* [‘to colour’]) are clearly reflective of the preschool context and specific to the population.

The vocabulary list may also be useful for purposes outside the realm of AAC. Representing words that are frequently used by Afrikaans-speaking Grade R learners, the list could be consulted in the development of formal and informal language and vocabulary assessments. It could also be consulted when developing intervention tasks targeting syntax and morphology, in an effort to choose familiar vocabulary that does not add a linguistic processing burden. However, the small and homogenous participant group has to be kept in mind, and caution should be exercised to avoid overgeneralisation of the results.

The current core vocabulary list was generated based on conversational samples from a small and homogenous group of participants. Since the way persons use language and vocabulary is intricately related to their age, as well as to the social and physical micro- and macro-context and the activity settings in which they find themselves, collecting further spoken Afrikaans language samples from persons of different age groups, various geographical regions, and during various communication situations could help to establish a more representative core vocabulary list. Furthermore, studies are needed to determine if design conventions for core–fringe based AAC systems that have been developed for other languages (e.g., English systems such as Tobii Snap Core First^TM^,^1^ the Super Core grid set for Grid 3^TM^,^2^ and the Pixon^TM^ communication boards^TM^^3^) are appropriate for Afrikaans core-fringe-based systems. Research to determine effective methods for introducing the use of such systems to persons in need of AAC and their partners is also urgently needed.

## Conclusion

This study contributes to the growing body of research aiming to support professionals and other team members with the challenging task of selecting the appropriate vocabulary for not (yet) literate individuals. The identified list of Afrikaans vocabulary core words can be used as a source for guiding vocabulary selection for young children in need of an Afrikaans AAC system. Specifically, the variety and proportion of content and structure words, as well as words from different parts of speech found in the core vocabulary, point to the necessity of including such a variety on a system that is intended to encourage novel utterance generation.

## Footnotes

1 Tobii Snap Core is a product of Tobii Dynavox of Pittsburgh, PA, USA, https://www.tobiidynavox.com

2 The Grid 3 is a product of Sensory Software International Limited of Malvern, England, https://www.sensorysoftware.com

3 Pixon is a product of Prentke Romich Company of Wooster, OH, USA, https://www.prentrom.com

## Appendix 1

### Afrikaans core vocabulary list

- a [CS]
- aan
- af
- ag
- agter
- ah
- al (adverb)
- al (numeral)
- alles
- almal
- altyd
- amper
- AN
- and [CS]
- ander
- as
- askuus
- asseblief
- baie
- be [CS]
- begin
- bietjie
- blok
- blou
- boek
- bou
- breek
- bring
- bruin
- by
- CN
- come [CS]
- cool [CS]
- daai
- daar
- dan
- dankie
- dat
- die
- dié
- dieselfde
- ding
- dink
- dis
- dit
- doen
- dogter
- drie
- een (pronoun)
- een (numeral)
- een_twee_drie
- eers
- eerste
- eet
- ek
- en
- gaan (auxiliary verb)
- gaan (lexical verb)
- gebruik
- gee
- geel
- go [CS]
- goed
- gooi
- gou
- groen
- groot
- haar
- hallo
- hand
- hei
- hele
- help
- het (auxiliary verb)
- het (lexical verb)
- hier
- hierdie
- hierso
- hmm
- hoe
- hoekom
- hom
- hoog
- hoor
- hou
- huh
- huh_uh
- huis
- hulle
- hy
- I [CS]
- iemand
- iets
- I’m [CS]
- in
- inkleur
- is [CS]
- is
- it [CS]
- ja
- jou
- juffrou
- julle
- jy
- kan
- keer
- ken
- kies
- kind
- klaar
- klas
- klein
- kleur
- kom
- kort
- kos
- koukie
- kry
- kyk
- laat
- lekker
- lelik
- loop
- los
- lyk
- ma
- maak
- maar
- maat
- mag
- mamma
- man
- meer
- mens
- met
- mhmh
- mmh
- mmm
- moenie
- moet
- mooi
- my [CS]
- my
- myne
- ’n
- naam
- nè
- nee
- net
- nie
- niemand
- niks
- nog
- nou
- now [CS]
- oe
- of
- oh
- om
- on [CS]
- ons
- ook
- op (adverb)
- op (preposition)
- oranje
- oukei
- ouma
- ow
- pasop
- pers
- prent
- reg
- regtig
- rooi
- ry
- saam
- sal
- se
- sê
- seer
- seun
- sien
- sirkel
- sit
- slang
- s’n
- so
- soek
- sommer
- soos
- speel
- staan
- sulke
- sussie
- swart
- sy (possessive pronoun)
- sy (personal pronoun)
- tannie
- te
- teken
- tel
- the [CS]
- this [CS]
- TN
- to [CS]
- toe
- tot
- twee
- uit
- um
- val
- van
- vandag
- vat
- vier
- vinnig
- vir
- voel
- voor
- waar
- wag
- wanneer
- want
- wat
- water
- watter
- weer
- weet
- weg
- werk (noun)
- werk (verb)
- wie
- wil
- wit
- word
- wys
- yay
- yes [CS]
- you [CS]

CS, Code Switch; AN, adult name; CN, child name; TN, teacher name.

Note: Only the uninflected root forms of nouns, verbs, numerals, pronouns and adjectives are given as all inflected forms were counted under the root form.
